# Procurement of Category 2 Vaccines in China

**DOI:** 10.3390/vaccines7030097

**Published:** 2019-08-23

**Authors:** Jian-Lin Zhuang, Abram L. Wagner, Megan Laffoon, Yi-Han Lu, Qing-Wu Jiang

**Affiliations:** 1 Department of Epidemiology, School of Public Health, Key Laboratory of Public Health Safety (Ministry of Education), Fudan University, Shanghai 200032, China; 2 Office of General Affairs and Emergency Management, Centers for Disease Control and Prevention of Changning District, Shanghai 200051, China; 3 Department of Epidemiology, School of Public Health, University of Michigan, Ann Arbor, MI 48109, USA; 4 Department of Environmental Health Sciences, School of Public Health, University of Michigan, Ann Arbor, MI 48109, USA

**Keywords:** China, vaccine procurement, vaccine economics

## Abstract

Internationally, vaccine pricing is relatively opaque, although many low- or lower-middle-income countries belong to international consortiums that jointly procure vaccines. China procures vaccines domestically, and vaccines that require payment from the public (“category 2 vaccines”), have undergone several regulatory changes over the past 15 years. This study aims to describe the vaccine procurement method changes in China since 2005 and to analyze how the procurement method impacted vaccine price. This review of vaccine procurement reforms found that a shift to provincial-level Group Purchasing Organizations after 2016 was accompanied by an increase in most prices. There was more variability in vaccine prices across provinces for vaccines with only one supplier, and these vaccines have a higher price than what is found in United Nations Children’s Fund (UNICEF)-supported countries. China’s current procurement system for non-mandatory vaccines leaves these vaccines costing several-fold more than in other countries, and in particular those supported by Gavi, the Vaccine Alliance. Exploring a variety of methods to reduce vaccine purchase prices will not only directly benefit the general population, but also the government, as they aim to implement more programs to benefit public health in a cost-effective manner.

## 1. Introduction

Vaccines are generally thought to be cost-effective interventions to prevent disease [1], particularly for those pediatric immunizations recommended by the World Health Organization (WHO) [2]. However, recently introduced vaccinations like the pneumococcal conjugate vaccine (PCV) and the human papillomavirus (HPV) vaccine are highly expensive [3,4,5], although they may still be cost-effective for use in middle-income countries like China, especially in wealthier regions of the country [6,7,8].

As many countries continue to prioritize vaccine affordability, an increasing amount of attention has been directed toward price transparency. In comparison with other life-saving medication, vaccines are relatively opaque in their pricing [9]. Manufacturing complexity, potential for medical patents, market competition and other production factors have all influenced the cost of a single vaccination; however, the overall purchasing price appears to be heavily reliant on the procurement avenue [10].

China divides vaccines into two categories, which have different pricing strategies, but which are both offered at community health centers across the country. As a result of governmental centralized purchasing, category 1 vaccinations are free of charge. In contrast, under China’s current policies, category 2 vaccines must be purchased by the public, and thus have a price barrier, particularly for lower-income families. Coverage of category 2 vaccines is lower than category 1 vaccines [11], although one survey throughout China did find that 61.41% of children had received a category 2 vaccine [12]. Better understanding the existing challenges in the financial and regulatory environment around category 2 vaccines is the first step to creating a better platform for parents to be able to buy category 2 vaccines.

Government regulations about vaccines are tied to the administrative divisions of China. China is divided into 34 provincial-level administrative units (including special autonomous regions, provincial-level municipalities, and special administrative regions), which in turn are made up of counties (termed districts in some urban settings). There are Centers of Disease Control and Prevention (CDCs) at the national, provincial, and county levels, with oversight of various activities including surveillance, disease prevention, and health promotion in their catchment area [13].

In 2005, the Chinese government issued the original “Regulations on the Administration of Vaccine Circulation and Vaccination” [14], and later updated it in 2016 [15] to adjust for changes in procurement methods of category 2 vaccines. Prior to the 2005 regulations, vaccines were distributed in a tiered fashion: lower-level CDCs would purchase their vaccines from the higher-level CDC which would then sell and distribute the vaccines to the lowest tier to finally be utilized for inoculation. This will be referred to as the CDC-tiered system.

The new regulations in 2005 turned this system on its head by directing the base tier to independently purchase vaccines directly from qualified distributors [14], and hence the period between 2005 and 2016 is referred to as the independent purchasing system. The system shifted again in 2016 as individual provinces were directed to develop Group Purchasing Organizations (GPO), allowing county-level CDCs to purchase and distribute vaccines within their jurisdiction [15]. This system will be referenced as the Provincial GPO system.

Vaccine procurement methods vary globally, ranging from municipal to international purchasing systems. The United Nations Children’s Fund (UNICEF) was originally established by the United Nations to provide emergency healthcare and food to mothers and children affected by World War II. They primarily obtain their vaccine supplies through Gavi, the Vaccine Alliance. Established in 2000, Gavi utilizes a collective bidding system based on the aggregate demand of the world’s most impoverish countries, allowing them to establish long-term contracts with manufacturers and make large-scale purchases at a lower price [16]. Gavi then engages participating counties in a co-financing program in order to increase the vaccine rate among low- and lower-middle-income countries. China currently does not participate in these multinational vaccine procurement efforts, but as countries economically develop past the point at which they can be supported by Gavi, it will be useful for them to consider what procurement method is most useful for their own circumstances.

At present, there is a deficit in reports concerning the relationship between the price and procurement method of category 2 vaccines. This study aims to describe the vaccine procurement method changes in China since 2005 and to analyze how the procurement method impacted vaccine price. This analysis will provide more transparency on vaccine pricing methods.

## 2. Materials and Methods

### 2.1. Data Sources

We obtained bidding information about category 2 vaccines from various provincial and municipal websites within China. We used six provinces or provincial-level municipalities as examples of the diversity in prices: Guangdong [17], Shanghai [18], Tianjin [19], Jiangxi [20], Chongqing [21], and Xinjiang [22]. We also abstracted data of vaccine prices published on the UNICEF website [23,24], from the US CDC Vaccines for Children program [25], and from European lower- and upper-middle-income countries [9]. All data are publicly available from these websites.

We considered the following vaccines (which were all category 2 vaccines in China at the time of data abstraction): enterovirus 71 (EV71) vaccine, *Haemophilus influenzae* type b (Hib) vaccine, hepatitis A (HepA) vaccine, hepatitis E (HepE) vaccine, human papillomavirus (HPV) vaccine, influenza vaccine, meningococcal ACYW135 vaccine, 13-valent pneumococcal conjugate vaccine (PCV13), 23-valent pneumococcal polysaccharide vaccine (PPSV23), rotavirus vaccine, and varicella vaccine. Information on several combination vaccines was available: a diphtheria-tetanus-acellular pertussis (DTaP)-Hib vaccine, a DTaP-Hib-inactivated polio vaccine (IPV) (note that this “pentavalent” combination in China differs from the one worldwide which is a DTaP-Hib-hepatitis B (HepB) vaccine), and a meningococcal AC-Hib vaccine.

### 2.2. Analytical Methods and Comparisons

We first descriptively review the three systems for procuring category 2 vaccines in China and their political and economic circumstances. Subsequently, using a standard set of vaccines, we compare prices across 4 circumstances:

(a) Provincial-level GPO purchasing vs. CDC-tiered purchasing;
(b) Provincial-level GPO purchasing vs. Independent purchasing;
  Between-province GPO purchasing differences: vaccine prices from six provinces or provincial-level municipalities in China (Guangdong, Shanghai, Tianjin, Jiangxi, Chongqing, and Xinjiang) were compared.
(c) Provincial-level GPO purchasing vs. International collective purchasing: vaccine pricing from UNICEF and other organizations was compared to Chinese provincial-level GPO vaccine prices.

We compare these prices in graphical and tabular format. A Pearson’s correlation coefficient (r) was computed to examine the correlation between the lowest/highest procurement vaccine price in 2016 and the difference between the GPO vaccine price in 2017 and other procurement methods in 2016. Data were analyzed in IBM SPSS Statistics 20.0.0 (IBM SPSS Inc., Chicago, IL, USA). A *p*-value of <0.05 was considered statistically significant.

### 2.3. Ethical Approval

This study did not include human subjects and so we did not obtain ethical approval.

## 3. Results

**Figure 1:**
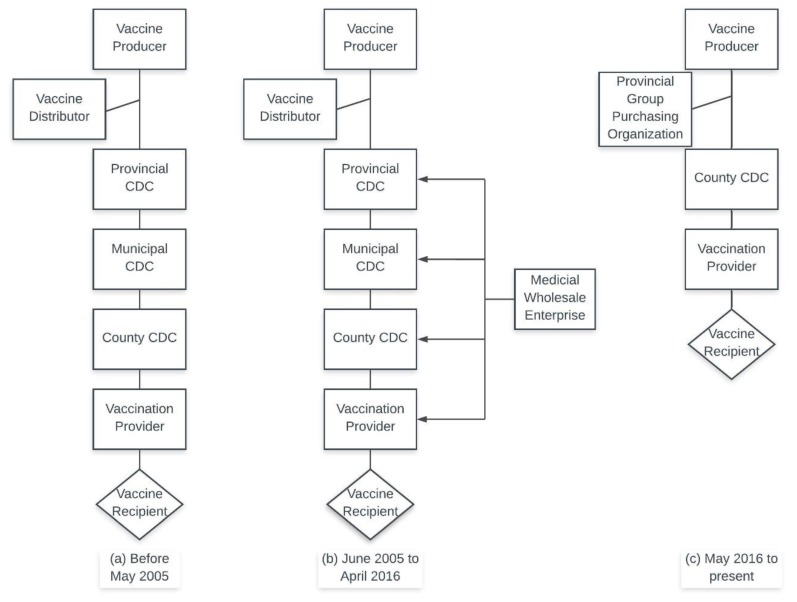
Schematic diagram of formal distribution channels of category 2 vaccines in China: (**a**) Centers for Disease Control and Prevention (CDC)-tiered system, (**b**) independent purchasing system, and (**c**) Group Purchasing Organization (GPO) system.

Prior to the 1980s, the Chinese economy operated as a centrally planned economy under government direction. As such, public health agencies relied on the government to provide operational funding, including freely provided vaccines. However, since Chinese economic reforms in the 1980s, this financial burden shifted to public health agencies. In order to compensate for limited investment into the public health sector, the government allowed different levels of public health departments and vaccination providers to charge a certain percentage of vaccine management and vaccination costs, including management fees, transportation costs, storage costs as well as the institutional and personnel costs needed to run public agencies or vaccination clinics [26]. As shown in Figure 1a, beginning with the vaccine producer, vaccines would be purchased down each tier of the CDC until the vaccination provider is reached. At each point, the price of the vaccines would increase (with a markup rate varying greatly across levels and across areas of China) [27,28]. Ultimately, the vaccinee would bear the cost of these mark ups.

Between 2005 and 2016, China allowed qualified pharmaceutical wholesale companies to sell vaccines directly to individual inoculation points at a negotiated price [14]. This process lacked adequate constraints and oversight in some area, and it has been thought to be responsible for the case of illegal sales of vaccines in Shandong where the improper storage of vaccines led to hundreds of thousands of faulty vaccines being administered to children [29].

In order to deal with problems in the field of vaccine procurement, the 2016 update vastly improved the procurement and pricing system [15]. Currently, a provincial-level GPO is used for county-level CDCs to purchase the vaccines and then to distribute them to vaccination providers in the region. This regulation effectively eliminates price differences at different levels of CDCs and vaccination providers, leading to uniform pricing within each province. Instead of markup rate, different service fees are charged in different provinces such as 20 renminbi (RMB) ($2.90)/dose in Jiangsu province and 28 RMB ($4.10)/dose in Zhejiang province [30,31]. Under the provincial GOP system, vaccine providers provide bids once a year, and upon completion of negotiations within each province, the prices are officially announced to the public.

Figure 1 shows the changes in category 2 vaccine procurement over time.

### 3.1. Provincial-Level GPO Scheme vs. CDC-Tiered Purchasing

**Figure 2:**
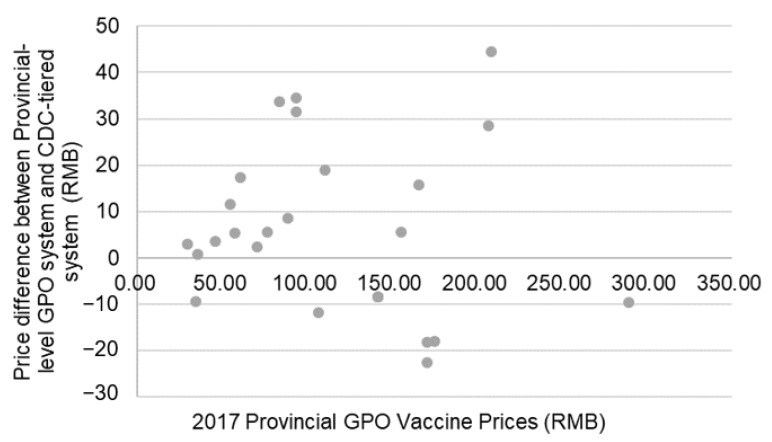
Difference in prices between vaccines procured through the provincial GPO system and the difference between the provincial GPO system and the CDC-tiered system.

Before shifting to the Provincial GPO method in 2016, Shanghai utilized the CDC-tiered method to obtain its vaccines. According to the prices published by the Shanghai government [18], among the 26 vaccines included in the 2016–2017 procurement catalogue, 17 increased in price by an average of 22.06% or 16 RMB ($2.30); and 9 decreased by an average of 12.70% or 9 RMB ($1.30). There was no significant correlation between GPO purchasing price and the change between the CDC-tiered price and the GPO purchasing price. Figure 2 shows the price and price changes of these 26 vaccines.

### 3.2. Provincial-Level GPO Purchasing vs. Independent Purchasing

In 2005, independent procurement from qualified pharmaceutical wholesale companies was legalized. A report published by the Guangdong Pharmaceutical Exchange Center included relevant pricing information such as a price summary of vaccines before the change in 2016 [17]. A total of 63 vaccines were listed in the Guangdong procurement catalogue.

**Figure 3:**
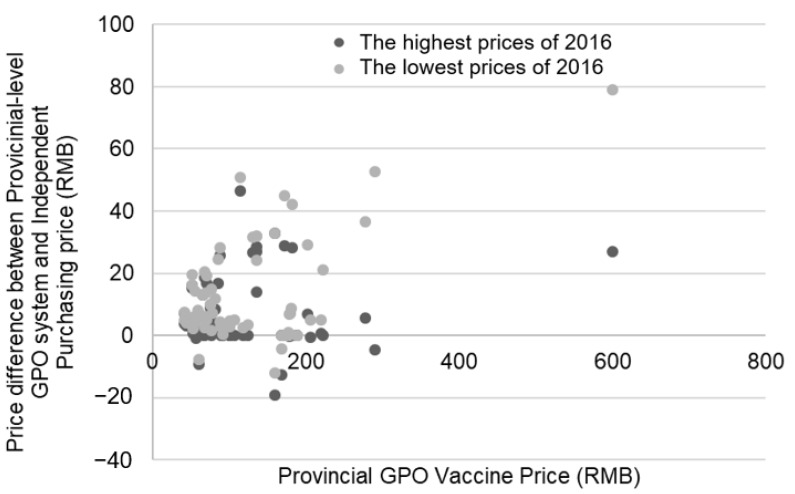
Difference in prices between vaccines procured through the provincial GPO system and the difference between the provincial GPO system and the Independent Purchasing price. The darker dot represents the price difference between the highest prices of 2016 and the price in 2017. The lighter dot represents the price difference between the lowest prices of 2016 and the price in 2017.

The catalog includes varying procurement prices per unit of diverse vaccine products among different regions, which meant that every kind of vaccine product had a highest and lowest procurement price. Figure 3 shows the price variances between both the highest and lowest purchasing price per unit of 2016 and the provincial GPO vaccine price of 2017. When examining the highest purchasing price per unit in 2016, 36 vaccines increased by an average of 16.94% or 13 RMB ($1.90); 19 vaccines did not change in price; and 8 decreased by an average of 4.82% or 6 RMB ($0.90). When examining the lowest purchasing price per unit in 2016, 54 vaccines increased by an average of 8.72% or 16 RMB ($2.30); 6 vaccines remained the same; and 3 decreased by an average of 11.73% or 8 RMB ($1.20).

To determine the relationship between 2016 and 2017 vaccine prices (2017 being the first year of the GPO scheme), we calculated two correlation coefficients, one between the lowest price of 2016 and the 2017 price, and one between the highest price of 2016 and the 2017 price. Overall, the correlation for the lowest price (r = 0.372, *p* = 0.003) in 2016 was significant whereas the result for the highest price (r = −0.051, *p* = 0.691) in 2016 demonstrated no significant correlation. Accordingly, there was a significant increase in price between 2016 and 2017, but only in regard to the lowest price vaccines in 2016.

### 3.3. Between-Province GPO Purchasing Differences

Data on the winning bids for vaccine prices in 2017 were obtained from governmental publications from Shanghai, Guangdong, Tianjin, Jiangxi, Chongqing, and Xinjiang. The vaccines and their prices are shown in Table 1. The first six listed vaccines (DTaP-Hib, DTaP-IPV, Hib, MenAC-Hib, rotavirus, PCV13, and HepE vaccines) had only one supplier. The prices of these vaccines varied within 5%. The other 7 vaccines could be obtained through multiple suppliers. Price variations for this latter group of vaccines are comparatively larger, and the effect of market competition is apparent.

### 3.4. Provincial-Level GPO Purchasing vs. International Collective Purchasing

Table 2 is a comparison of vaccine purchases published online by UNICEF and Shanghai. Overall, China’s category 2 prices are higher than those of UNICEF and Europe and more comparable to those from the Vaccines for Children program in the US.

The data from Table 2 show that vaccines purchased from a single supplier, as is done in China for the vaccines for rotavirus, PCV13 and DTaP-Hib, come with a higher premium cost. For example, the price of PCV13 in regions of China can be more than 30 times the minimum price of UNICEF. Additionally, in the case of the Hib, influenza, HPV and varicella vaccines, countries with multiple domestic suppliers experience increased competition and lower prices.

## 4. Discussion

Historically high vaccine prices have pushed reforms in vaccine procurement to the forefront. For example, the WHO launched the V3P (Product, Price, Procurement) project in 2014 to increase price transparency [10]. This review of vaccine procurement reforms in China found that a shift to provincial-level Group Purchasing Organizations (GPOs) after 2016 was accompanied by an increase in most vaccine prices. There was more variability in vaccine prices across provinces for vaccines with only one supplier, and these vaccines have a higher price than what is found in UNICEF-supported countries. In Guangzhou, the vaccine price difference (from before to after the GPO scheme) was weakly correlated with the lowest procurement price but not the highest procurement price of 2016. This result may indicate that in the negotiations for the GPO price in 2017, the highest price from 2016 was used.

### 4.1. Changes in Category 2 Vaccine Procurement in China

Prior to 2005, vaccines were procured through the CDC structure, which had several flaws. As with all commodities, the greater the number of links in the supply chain, the greater the ultimate consumer price. For example, the category 2 vaccine prices published by the Hangzhou Municipal CDC showed a markup of about 7.5% for all types of vaccines [32]. According to a review conducted by Ma et al. [33], the cumulative markup rate for each intermediate distribution link across 31 different category 2 vaccines added up to 45.58%, accounting for almost half of the final retail price. Simultaneously, it could be argued that this system had limited effectiveness to manage vaccine problems because of the large number of links.

After April 2016, the procurement method for China’s Category 2 vaccines shifted to the use of GPOs [15]. Compared with the previous mechanisms (procurement through the CDC in a tiered approach and through market-based independent procurement), there are several clear advantages. First, the development of GPO standards led to a list of registered vaccines and may have diminished the use of fraudulent products. Second, a unified source of procurement eliminates the possibility of illegal suppliers, and, third, the increased price transparency diminishes regional price differences.

For the highest priced vaccines, increased market competition, particularly from domestic manufacturers, could provide some relief. Currently, the HPV vaccine, the DTaP-IPV-Hib vaccine and PCV13 are all priced above 500 RMB ($73), limiting their economic accessibility, especially outside wealthier areas in cities or in eastern China. PCV13, as a specific example, is 704 RMB ($102.00) in the Shanghai GPO. According to V3P information [10], the average price of PCV13 in high-income countries was $51.60 (356 RMB), meaning that the purchase price for PCV13 in China was about 1.7 times higher. In contrast, the Hib and influenza vaccines are produced by multiple manufacturers, and this market competition has resulted in comparable or lower prices than those found in the US public sector.

Vaccine prices could change in China based on demand and production. In general, a decline in commodity prices corresponds with an increase in consumer demand [34]. In China, the per capita use of category 2 vaccines is markedly higher in the economically developed areas in Eastern China as opposed to western provinces [35]. Excessive pricing may be a significant obstacle for these lower-income areas. It follows that lowering the price of the category 2 vaccines may lead to an increase in vaccine uptake among less affluent families. Concomitantly, domestic vaccine production in China is booming, and increased competition could drive down prices. Vaccine production from China could have a massive impact not only on the Chinese population, but also worldwide [36].

We note that the Chinese government has recently implemented new legal changes to vaccine safety, in its “Law of Vaccine Management in the People’s Republic of China” which will come into effect on 1 December, 2019 [37]. This law introduces several new regulations, including increased fines on producers and distributors of defective vaccines and compensation for individuals with adverse events following immunization. However, the regulation continues to stipulate that the procurement of category 2 vaccines is through provincial-level GPOs. Therefore, it is expected that this new law will not substantively impact vaccine prices.

### 4.2. Global Comparisons of Vaccine Prices

The WHO’s V3P project collects data from WHO member countries and international organizations such as UNICEF on vaccine prices, contract length, and purchase quantity. The project found that price varied across numerous factors including quantity of purchase, purchasing method, payment method, contract length, and country income. Vaccine prices also varied widely by region [10]. It is important to recognize that vaccine prices are positively correlated with a country’s per capita income level [38].

According to UNICEF reports, UNICEF provides at- or near-cost vaccines to 40% of the world’s children, primarily through Gavi [24]. By engaging in large-scale collective bidding and price conversion, and by instituting a tiered pricing scheme where low-income countries pay the least for vaccines, Gavi is able to achieve the lowest supply price in the world [16]. For example, for the 10-valent and 13-valent PCV, the weighted average price in 2015 for Gavi-sponsored low-income countries was $4.18, whereas for other low-, middle-, and high-income countries, it was $15.44, $20.51, and $51.60 USD, respectively—an up to 10-fold price difference [24].

According to vaccine price data from the WHO European Region [9], the greater the market competition, the lower the vaccine price; for example, the influenza vaccine has many producers and is in high demand. Any single country which independently purchased vaccines, such as China, paid a significantly higher price. Vaccine prices in China were 5–20 times more expensive than vaccines available from UNICEF, and more often approached public sector vaccine costs from the US [25]. Vaccine prices in China were also higher than middle-income countries in Europe [9]. We note that countries in Europe which have implemented programs to jointly pool vaccine procurement across borders to increase purchase volume have achieved some preliminary success in reducing costs [38].

From a production standpoint, as purchase quantity increases, production costs decline. Clendinen et al. [39], in a study examining manufacturers which produce and supply the HPV vaccine to developing countries, found that with the increase in supply quantity, the production price of a single dose vaccine decreases sharply, potentially increasing the profit margin on the 4-valent HPV vaccines by $0.50–0.60 USD per dose (equivalent to 3–4 RMB).

### 4.3. Strenghts and Limitations

The results of this study have several limitations. Public access to relevant government data is subject to restrictions, ultimately limiting the scope of the study. Additionally, data models related to the CDC-tiered system and independent procurement were restricted to a single province or municipality, limiting the generalizability of the findings. Of note, however, is that after the implementation of the 2016 regulations, the purchases prices vary little by region and have not exemplified a downward trend, indicating that the single sources of data we used could be applicable to other areas. While similar studies have been conducted on general medication prices, this is the first comparative study of vaccine pricing based on procurement models. Changes in prices could be due to many reasons not examined in this study, including volume purchased, supply vs. demand, inflation, and the number of vaccine producers.

## 5. Conclusions

China’s current procurement system for non-mandatory vaccines leaves these vaccines costing several-fold more than in other countries, and in particular those supported by Gavi. Gavi has played a driving role in directing the vaccine market to lower pricing using bulk purchasing for many different countries at one time. While Gavi‘s model is not completely applicable in China, it can serve as a useful reference in directing China’s future vaccine pricing and procurement systems. In addition to modeling pooled demand, Gavi also applies other economic tools to lower prices such as the use of long contracts to secure a steady supply, encouragement of market competition, and strict quality management. Exploring a variety of methods to reduce vaccine purchase prices will not only directly benefit the general population, but also the government as they aim to implement more programs to benefit public health in a cost-effective manner.

## Figures and Tables

**Table 1:** Comparison of vaccine prices in different provinces of China, 2017.

Vaccine	Guangdong	Shanghai	Tianjin	Jiangxi	Chongqing	Xinjiang	Highest/Lowest Price
DTaP-Hib	278.00	285.50	274.53	275.00	-	308.00	1.12
DTaP-IPV-Hib	600.00	604.50	-	599.00	599.00	-	1.01
MenAC-Hib	222.90	221.50	216.00	-	-	226.00	1.05
Rotavirus	172.00	173.5	-	172.00	-	172.00	1.01
PCV13	-	703.50	698.00	698.00	-	698.00	1.01
HepE	160.00	165.50	-	160.00	-	-	1.03
Hib	84.98	91.46	91.71	80.66	95.27	85.86	1.18
Varicella	134.75	144.95	138.93	136.54	136.00	140.63	1.08
PPSV23	192.25	197.50	189.61	191.00	200.00	-	1.05
EV71	178.00	193.50	184.26	174.67	168.00	184.67	1.15
HepA	116.31	118.88	118.78	130.50	151.00	105.37	1.43
MenACYW135	60.88	64.83	64.00	56.45	56.00	64.71	1.16

Abbreviations: DTaP—diphtheria-tetanus-acellular pertussis; EV71—enterovirus 71; HepA—hepatitis A; HepE—hepatitis E; Hib—*Haemophilus influenzae* type b; IPV—inactivated polio vaccine; Men—meningococcus; PCV13—13-valent pneumocococcal conjugate vaccine; PPSV23—23-valent pneumococcal polysaccharide vaccine.

**Table 2:** Comparison of vaccine prices from different organizations.

Vaccine	UNICEF (2017)	Europe MICs (2013)	US CDC Public Sector Cost (December 2017)	Shanghai GPO (2018) ^3^
Hib	23 RMB $3.40 ^1^	$4.03	$9.46–$12.79	71–107 RMB $10.20–15.40
Rotavirus	14–22 RMB $2.00–3.20	$2.46–2.96	$69.12–91.05	174 RMB $25.10
PCV13	23 RMB $3.30	$3.53–41.28	$126.97	704 RMB $102.00
HPV ^2^	31–32 RMB $4.50–4.60	$53.97–93.40	$154.28	587–804 RMB $84.90–116.40
Influenza	-	$2.73–14.29	$13.55–15.68	26–56 RMB $3.70–8.00
Varicella	-	$17.35	$92.72	142–155 RMB $20.50–22.50
DTaP-HepB-Hib	6–10 RMB $0.80–1.40	-	-	-
DTaP-IPV-Hib	-	$7.98–16.56	$56.74	600 RMB $87.00

Abbreviations: DTaP—diphtheria-tetanus-acellular pertussis; EV71—enterovirus 71; HepA—hepatitis A; HepE—hepatitis E; Hib—*Haemophilus influenzae* type b; IPV—inactivated polio vaccine; Men—meningococcus; MIC—middle-income countries; PCV13—13-valent pneumocococcal conjugate vaccine; PPSV23—23-valent pneumococcal polysaccharide vaccine; ^1^ Price of the stand-alone Hib vaccine in 2011. After, Hib was only available in combination with other vaccines; ^2^ Range reflects 2- and 4-valent HPV vaccines; ^3^ The data of 2018 were used due to the introduction of the HPV vaccine in this year.
